# The Journal of Neurology and Psychopathology

**Published:** 1920-08-28

**Authors:** 


### "The Journal of Neurology and Psychopathology."

We have received a copy of the publication bearing the above title, and would draw the attention of those interested in these subjects to the extremely valuable new quarterly under the joint editorial direction of the following: Dr. S. A. Kinnear Wilson, Dr. T. Graham Brown, Dr. R. M. Stewart, Dr. Bernard Hart, Dr. Henry Devine, Dr. Maurice Nicoll, and Dr. Carey F. Coombs. The greater part of the journal is devoted to first-class original papers, followed by editorial sections, and the remainder, apart from news items, consists of a particularly useful set of abstracts and comments upon the current contemporary literature the world over. Those responsible for the first number are to be heartily congratulated. The price is 30s. net per annum, or single copies at 8s. 6d. each net. Published by John Wright & Sons, Bristol.

